# Meteorological Dependencies in Ducks: Influence of Weather on Sex Ratio Bias and Hatchability

**DOI:** 10.1111/asj.70205

**Published:** 2026-06-24

**Authors:** Valeriy G. Narushin, Michael N. Romanov, Sabine Klein, Attila Salamon, Gianluca Manzoni, Darren K. Griffin, John P. Kent

**Affiliations:** ^1^ Independent Scientist Zaporizhya Ukraine; ^2^ School of Natural Sciences University of Kent Canterbury Kent UK; ^3^ Animal Genomics and Bioresource Research Unit (AGB Research Unit), Faculty of Science Kasetsart University Bangkok Thailand; ^4^ L. K. Ernst Federal Research Center for Animal Husbandry Dubrovitsy Moscow Oblast Russia; ^5^ Institute of Farm Animal Genetics Friedrich‐Loeffler‐Institut Neustadt Germany; ^6^ Department of Ethology ELTE Eötvös Loránd University Budapest Hungary; ^7^ Ballyrichard House Arklow Co. Wicklow Ireland; ^8^ Institute for Womens’ Health University College London London UK

**Keywords:** ambient temperature and other weather conditions, duck eggs, embryo mortality and sex ratio bias, hatchability, reproductive physiology and behavior

## Abstract

The feasibility of altering the sex ratio of hatching ducklings artificially can be an important economic factor in reducing production costs. As a preliminary investigation, associations between seasonal and weather factors and the secondary sex ratio in domestic ducks were examined to establish mechanisms that may determine the sex ratio of offspring. Over several years, a predominance of male hatchlings was observed, with an average bias of 51%–52%. There was no significant relationship found between the sex ratio shift and a particular meteorological season. A greater robustness was achieved by separately considering the time cycles of growth and decline in oviposition. Examining weather factors, i.e., average temperature, wind speed, and sea level pressure, we found significant relationships between these parameters and the sex ratio of hatching ducklings during periods of increasing egg production of their mothers. Wind force had the greatest influence on hatchability, to a greater extent during periods of decline in egg production. The decrease in hatchability of the duck flock was associated with increased wind speed. Our findings are crucial for further experimental evaluation of the sex ratio under controlled ambient temperature and wind strength.

## 1 Introduction

There is a vast amount of research data on the reproductive physiology and behavior of birds and, it seems that the mother bird, either intentionally or otherwise, invests in the sex ratio of hatchlings and regulates how many offspring of a particular sex should be born in a given period of time (e.g., Bradbury and Blakey 1998; Nager et al. 1999; Rutkowska and Cichoń 2002; Rutstein et al. 2004; Pryke and Rollins 2012; DuRant et al. 2016). While a reasonable assumption might be that this tendency exists only in wild bird species, it turns out not to be the case as such a phenomenon is also observed in domestic species. According to Müller et al. (2002), who investigated the potential reasons for sex ratio bias in domestic chickens, parental investment in males or females is anticipated to occur in proportion to parental raising capacities. Several studies considered the possibility of sex ratio alteration in Japanese quails; for instance, Pike and Petrie (2006) tried to change it by manipulating the levels of testosterone, 17β‐estradiol, and corticosterone. This led to the observation that fecal concentrations of the main avian stress hormone, corticosterone, were highly associated with the offspring sex ratio, and experimentally raised corticosterone levels caused significantly female‐biased sex ratios at laying. Navara (2013a) supposed that hormones are likely to play a role in the adjustment of sex ratio in vertebrates and specified the influence of steroid hormones in birds (Navara 2013b). In a recent study, Navara et al. (2023) attempted to change the sex ratio in quails due to slowing follicle growth rates, but their efforts were unsuccessful. The authors recommended that this line of investigation be continued, focusing more on the components of the yolk immediately surrounding the germinal disc just before ovulation to see if mediators that build up in the yolk are instead what causes sex ratio skews in birds.

Perhaps the largest number of researchers in this area have concentrated their efforts on domestic ducks ( *Anas platyrhynchos* ). First of all, these studies were related to comparing the proportion of males to females (M:F) during fertilization, i.e., *primary* sex ratio, with the proportion of males seen in a population of day‐old ducklings, i.e., *secondary* sex ratio (e.g., Ericsson and Ericsson 1998). In a study carried out on several crosses of domestic ducks, Batellier et al. (2004) showed that secondary sex ratio change can occur in the course of egg incubation. Whereas the M:F ratio of embryos approached 1 during the first incubation days, the quantity of males after hatching clearly exceeded that of females. For practical purposes, it is the secondary sex ratio that is of greater interest. Considering that incubation losses are, for the most part, irreversible (Abd El‐Hack et al. 2019), it is highly desirable to establish the factors that influence the greater mortality of males or females during incubation. For example, according to Idahor et al. (2015), the sex of successfully hatched ducklings is influenced by the weight and shape of the eggs. If we assume an analogy between duck and chicken eggs, then, according to the results of studies by Wu et al. (2012), more females died from strains that produced smaller eggs, whereas more males perished from strains that produced larger eggs. That is, we can assume the existence of certain factors that induce hatching of more males or females. In the wild, females could manipulate sex ratio via timely differential embryo mortality (Alonso‐Alvarez 2006). Abd El‐Hack et al. (2019) reviewed a range of factors affecting duckling hatchability and suggested that there is a significant influence of both seasonal factors (Chowdhury et al. 2004; Awad 2013) and weather conditions (Farghly et al. 2018).

The ability to shift the sex ratio has enormous economic potential for the poultry industry. Although for ducks it is not as critical as, e.g., for chickens, in meat species, it would facilitate the production of a larger quantity of males that have a more developed ability to increase muscle mass. However, males are not in demand for commercial egg production. Again, for breeding flocks, 1 male to 5–8 females for meat‐type ducks and the sex ratio 1:6 to 1:8 for layer‐type ducks are typically used (e.g., Poultry 2024). That is, it can be argued that it is more economically advantageous to be able to create some preconditions for shifting the sex in one direction or another, depending on the production purpose of a flock, especially if this does not require significant additional costs. This raises the following questions. How can one achieve the desired sex ratio shift? What factors have the greatest influence on regulating it and achieving the desired shift artificially? Examples of studies in wild bird species demonstrated the dominance of the following basic conditions, which can also take place when keeping poultry: (i) seasonal changes (Appleby et al. 1997; Genovart et al. 2003; Li et al. 2008; Lehikoinen, Christensen, et al. 2008), (ii) egg incubation conditions (Eiby et al. 2008; DuRant et al. 2016; Kato et al. 2017), (iii) feeding stimuli (Reneerkens et al. 2002; Brommer et al. 2003; Rutstein et al. 2004), and (iv) weather factors (Boonstra 2004; Göth and Booth 2005; Pryke and Rollins 2012). Conditions (ii) and (iii) can be excluded as influential factors for the poultry industry practice in view of the established standard technological conditions and procedures for rearing and keeping birds. As for environmental factors (i) and (iv), they may promise some preferences in artificial sex ratio alteration. More precisely, it is the fourth point, of weather conditions, that deserves the most attention. On the other hand, a conditional “tuning” of the maternal body to a certain sex ratio shift depending on season (i) may negate the possibility of artificial sex ratio change using weather factors (iv). However, in a modern poultry house, one can create any economically advantageous environment (i.e., weather) conditions (e.g., Kaimujjaman et al. 2023; Godinho et al. 2025). Current demands may transform what appears premature and economically impractical today into a technologically justifiable requirement tomorrow.

Meteorological factors may influence the sex ratio of offspring with the mother bird's body optimally matching it to the expected quality of the rearing environment (Pryke and Rollins 2012). This feature is inherent in wild species, constantly facing weather challenges that affect the future welfare of their chicks. Notably, commercial duck crosses have not been subjected to such active selection as chickens. In this regard, certain adaptive mechanisms inherent in wild ducks remain effective in their domestic counterparts. Several studies have shown that in harsh winters, males of wild ducks survive to a greater extent (e.g., Brides et al. 2017), which may be due to many factors outlined in the work of Meissner and Witkowska (2023): (i) the cold tolerance, (ii) lower metabolic rate in males, and (iii) breeding advantage. However, the authors (Meissner and Witkowska 2023) found in their research the opposite trend of female prevalence of mallards ( *A. platyrhynchos* ) wintering in an urban habitat. It is possible that, in addition to the temperature, a number of other factors may have a noticeable impact.

Many researchers in this field agree that long‐term trends in duck sex ratios are not well‐studied, and information on this phenomenon among different populations and breeds remains limited (e.g., Pöysä et al. 2019; Wood et al. 2021; Homolková et al. 2024). In this regard, the goal of this study was to assess the influence of season and weather conditions on the shifted ratio of ducklings in a commercial duck flock. The latter was chosen as the object because of the following factors. Firstly, domestic ducks are not subject to strong breeding programs as chickens and because of that this species still has quite pronounced body features inherent in their wild counterparts. It is not without reason that many studies on sex ratio alteration in birds were carried out specifically on ducks, as we described above. Secondly, it is much easier for ducks to be kept free‐range throughout the entire year. Thirdly, unlike, for example, geese, ducks lay eggs all year round, allowing to judge adequately possible seasonal dependencies.

## 2 Materials and Methods

### 2.1 Ethical Note

The present study complied with the guidelines of the European Convention for the Protection of Vertebrate Animals used for Experimental and other Scientific Purposes (ETS No. 123, Strasbourg 1986) required to address any animal welfare issues arising from the study. The appropriate experimental design and procedures were employed to address any ethical implications. Accordingly, the proper study procedures were taken to minimize adverse impacts on, and not to compromise, the welfare of animals. No ducks used in the study were subject to euthanization, vivisection, or any other inhumane treatment. The best and humane practices of poultry husbandry and management were observed to reduce stress in animals. All ducks were alive and healthy after the study and used for further flock management. The proper housing, management, and incubation conditions were fully adhered to following the previous procedure descriptions for ducks (Salamon and Kent 2014, 2016). Specifically, the duck flock was maintained within a standard farm and treated using generally accepted and humane husbandry practices. During this study, experimental activities were performed that did not cause additional distress and disturbance to the birds and did not require ethical approval or bioethical oversight from relevant regulatory authorities. These activities included egg collection, productivity analysis, behavioral observation in the farm environment, and recording of existing parameters (weather influences) that did not induce any harm or anxiety to the birds.

### 2.2 Experimental Animals, Flock Management, and Egg Incubation

A commercial flock of Aylesbury ducks at Ballyrichard Farm (Arklow, Ireland; 52°50′5″ N, 6°7′49″ W) were investigated from January 2014 to September 2020. The total amount of ducks in the flock was ranging from 859 to 1141 during the observation period. The sex ratio in the flock was approximately 1 male per 6 females. This ratio was set up artificially during the formation of the herd in accordance with the recommendations available elsewhere (e.g., Poultry 2024). The ducks were housed in one shed unit (12.6 × 7 m) at night, released at 11:00 h (GMT) to an adjacent grass field with a water supply and had access to their houses with feed and water during the day. The sheds do not have controlled temperatures. The relatively temperature in the sheds was facilitated by their being approximately 3 m below ground at one end ensuring that the temperature is relatively constant not getting very cold in winter and not getting very warm in summer. Ducks were maintained on a natural daylight schedule, with additional electric light until 22:00 h to maintain a light schedule close to 16 h per day. Eggs were collected at 09:30 h and again at 11:00 h daily, washed and stored until setting at fortnightly intervals. The access to the field during the daytime enabled the assessment of the direct impact of both seasonal and weather variations on the duck flock. These conditions of growing and maintenance were fully in line with the EU Recommendation (Recommendation 1999) and other generally accepted recommendations concerning domestic ducks (as described elsewhere, e.g., Tereschenko et al. 2008; Shkurko et al. 2015; Antonov et al. 1991).

During the observation period, about 445,000 duck eggs were incubated, that is, an average of 63,500 per year. There is seasonal variation in egg production but the number of eggs set in the incubator was relatively constant throughout the year with a view to supplying customer needs. Temperature in the incubators was of approximately 37.2 °C. Egg storage temperature was maintained to not exceed 14 °C. Eggs were not stored for more than 14 days before incubation began. From each incubation set, the number of which was 843 for the entire observation period, a sample of about 5% of ducklings was randomly selected from the brooding pens on day 3 of life. After this, the ducklings were sexed in accordance with the procedure of Martin (1994). For each batch, the hatchability rate was recorded as the ratio of the number of ducklings obtained relative to the eggs set in the incubator. Thus, further analysis of the generated results was related to the secondary sex ratio, i.e., the number of individuals of both sexes that successfully completed the incubation process.

### 2.3 Weather Data

To analyze the possible influence of weather conditions on the main indicators of productivity of the parent flock of ducks, including sex ratio bias, hatchability, and egg production, we used archival meteorological data from the Weather Underground (2023) web service. The weather data archive from Arklow Weather Station IARKLO11, located in close proximity to Ballyrichard Farm, was used. Considering that hatching of ducklings occurs 28 days after setting eggs in the incubator, weather data were recorded for the month preceding the one during which incubation took place. For further analysis and searching for possible relationships, we selected all data categories that were available in Weather Underground (2023): (1) average temperature (*t*) in°C, (2) dew point in°C, (3) wind speed (*w*) in km/h, (4) gust wind in km/h, and (5) sea level pressure (*P*) in hPa. Thus, when selecting data, we were guided not by the possible biological connection of a particular weather factor with the bird's organism but, to a greater extent, by their presence in the period of time of interest to us.

### 2.4 Statistical procedures

The representativeness of each sample was calculated using the calculation formula for minimum sample from Cochran (1977) and an assumption on the margin of error (*E*). The acceptable value of *E*_max_ was taken at the level of 5% (0.05), according to Cochran (1977). Hence, we calculated the value of *E* and compared it to the permissible value of *E*_max_. The representativeness condition was that the calculated value of *E* should not exceed *E*_max_, i.e., 0.05. In this case, the calculation formula for minimum sample from Cochran (1977) was converted to the following:

(1) $$E=\frac{\left(N-n\right)\sigma_{y}^{2}}{\left(N-1\right)n}$$

where *N* is the total number of hatching eggs in the batch to be studied, *n* is the number of eggs in the respective sample, and *σ*_*y*_ is the standard deviation of the studied parameter that, in our case, corresponds to the sex ratio of ducklings in each of the samples.

The representativeness assessment demonstrated essential significance in the size of all samples. The value of *E* did not exceed 0.0008 that satisfied the condition of *E* ≤ 0.05 with a margin. Thus, it was possible to warrant the adequacy of the statistical processing of the results obtained from the observations conducted.

The strength of the relationship between parameters was assessed using the Pearson correlation coefficient *R*, with significance confirmed at *p* < 0.05. Experimental data were fitted to regression equations using the “non‐linear estimation” subfunction in STATISTICA 5.5 (StatSoft Inc./TIBCO, Palo Alto, California, United States). The validity of the resulting regression models was estimated using the coefficient of determination (*R* ^2^), with significance confirmed at *p* < 0.05. Multiple correlation was assessed by regression analysis using computational Microsoft Excel tools. Since regression analysis is performed with independent data that follow a normal distribution, certain checks should have been made beforehand. There was no possibility of adjusting natural weather factors during observations. Dependent variables were analyzed using flock means, which were updated annually. As a result, it can be concluded that the observations can be considered independent annual realizations. To confirm normal distribution, the Shapiro–Wilk test (Shapiro and Wilk 1965) was used. The following formula for calculating the Shapiro–Wilk criterion, *W*, was used:

(2) $$W=\frac{{\left(\sum_{i=1}^{n}x_{i}a_{i}\right)}^{2}}{\sum_{i=1}^{n}{\left(x_{i}-\bar{x}\right)}^{2}}$$

where *x*_*i*_ are the data values, *n* is the number of data, $\bar{x}$ is the mean value, *a*_*i*_ is the table value.

Comparison of the calculated *W* value (Equation 2) with the tabulated value *W*_*t*_ for *p* < 0.05 allowed us to conclude that the distribution is normal if *W* > *W*_*t*_.

Graphic visualization of experimental data was carried out in Microsoft Excel using a graphical editor.

## 3 Results and Discussion

### 3.1 Influence of Season on Sex Ratio

In the first stage, we estimated whether there is, somehow, and regardless of season, a chance to use sex ratio alteration in ducks for commercial purposes. Alternatively, we hypothesized that it was all as a result of the season, and there was no way to influence this process artificially. For this purpose, we considered the results obtained separately, both by the years studied and by seasons (Table 1).

**TABLE 1:** Summary data on the ratio of males (M) to females (F) for the studied years broken down by season.

Year/season^a^	M	F	M:F
**2014**			
Winter	688	671	1.025
Spring	1126	1139	0.989
Summer	588	553	1.063
Autumn	220	178	1.236
**Total for 2014**	**2622**	**2541**	**1.032**
**2015**			
Winter	439	418	1.050
Spring	1393	1180	1.181
Summer	983	922	1.066
Autumn^b^	—	—	—
**Total for 2015**	**2815**	**2520**	**1.117**
**2016**			
Winter	993	879	1.130
Spring	1686	1573	1.072
Summer	1239	1168	1.061
Autumn	912	944	0.966
**Total for 2016**	**4830**	**4564**	**1.058**
**2017**			
Winter	1362	1223	1.114
Spring	1939	1864	1.040
Summer	954	870	1.097
Autumn	981	1021	0.961
**Total for 2017**	**5236**	**4978**	**1.052**
**2018**			
Winter	1347	1392	0.968
Spring	1268	1110	1.142
Summer	986	876	1.126
Autumn	506	470	1.077
**Total for 2018**	**4107**	**3848**	**1.067**
**2019**			
Winter	2593	2533	1.024
Spring	1175	1062	1.106
Summer	964	799	1.207
Autumn	367	346	1.061
**Total for 2019**	**5099**	**4740**	**1.076**
**2020**			
Winter	1474	1368	1.077
Spring	1072	1050	1.021
Summer	2399	2313	1.037
Autumn	910	814	1.118
**Total for 2020**	**5855**	**5545**	**1.056**

^a^ The division of the seasons was as follows: winter means December–February; spring, March–May; summer, June–August; and autumn, September–November.

^b^ Due to technical problems, data for the autumn of 2015 could not be collected, which is why this period was excluded from the analysis.

The differences in M:F data between seasons for each year were not statistically significant.

**FIGURE 1:**
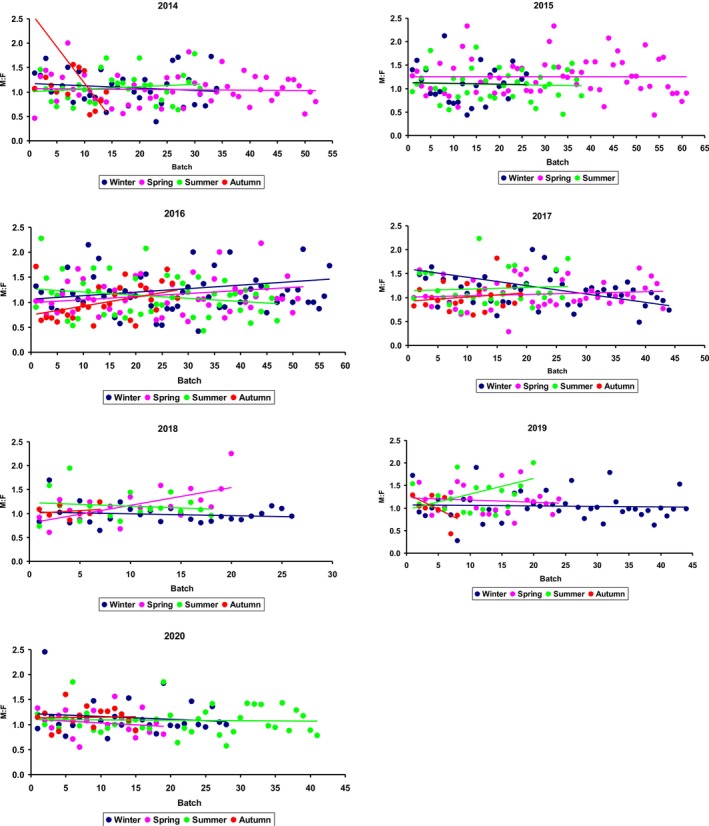
Ratio of males to females (M:F) as a result of duck egg incubation for each year (2014–2020) by season.

Besides the average numbers, it was interesting to investigate tendencies of the sex ratio changes during each of the seasons (Figure 1).

**FIGURE 2:**
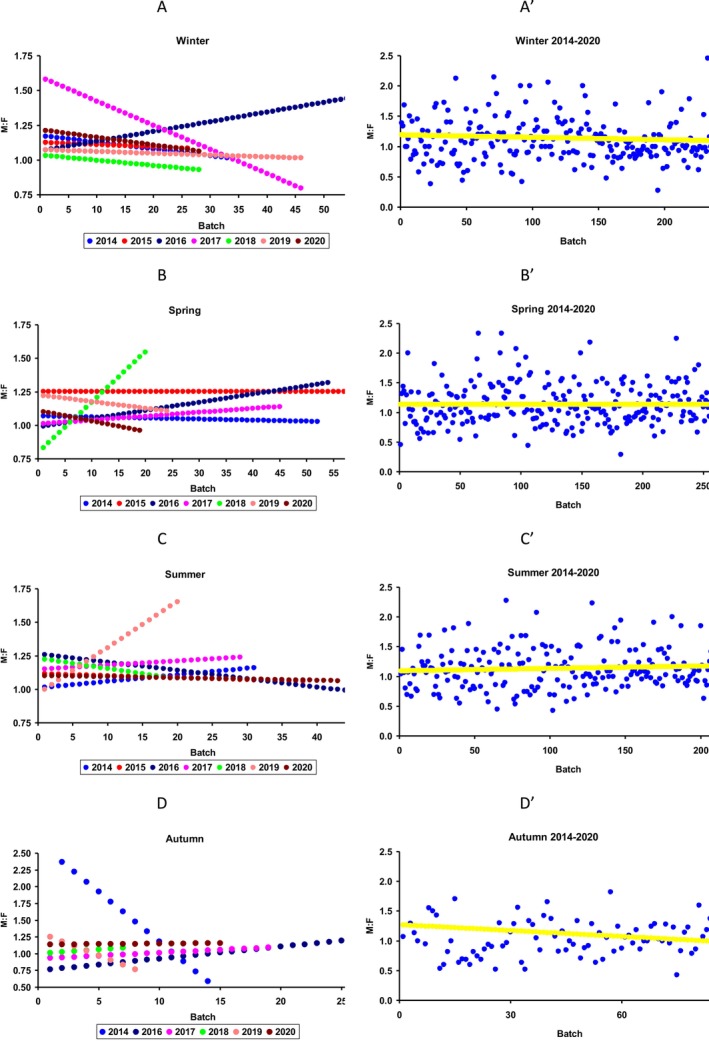
Summarized data of the ratio of males to females (M:F) as a result of duck egg incubation broken down by season for each year from 2014 to 2020 (A, B, C, D) and generalized over 7 years of observations (A′, B′, C′, D′).

Significant interannual variability of the obtained data did not allow us to draw a conclusion about any patterns, or at least trends, in changes in M:F values for each season. The only conclusion that can be drawn is, to a greater or lesser extent, a practically continuous bias of hatching males. We also made an attempt to catch this trend by summarizing the observation results for each of the four seasons over 7 years of observations (Figure 2). The summarized data on hatched males (M) and females (F) in each season for 7 years of observations, with the corresponding recalculation of their M:F ratio, are presented in Table 2.

**TABLE 2:** Data on the ratio of males (M) to females (F) by season for 7 years of observations.

Season	M	F	M:F
Winter	8896	8484	1.049
Spring	9659	8978	1.076
Summer	8113	7501	1.082
Autumn	3896	3773	1.033
**Total for 2014–2020**	**30,564**	**28,736**	**1.064**

The significance of the differences between the obtained data by seasons for each year (Figure 2A, B, C, D) and the total data by seasons, but for all 7 years of observation (Figure 2A′, B′, C′, D′), was assessed by their difference according to *t*‐test. As a result, for the winter season, the data by year were significant (*p* < 0.05) with the exception of 2015. Spring results showed significant differences (*p* < 0.05) with the exception of 2016. The summer period was significant (*p* < 0.05) with the exception of 2017. Data for the autumn were somewhat truncated due to the lack of information for 2015; however, differences compared to the average values were significant (*p* < 0.05) with the exception of 2018 and 2019.

The summary of M:F data again did not allow us to determine the general trend in this indicator. Any season in different years showed both a rapid increase in the sex ratio and a similar decline (Figure 2A, B, C, D). Summarized data for all seasons over 7 years of observation did not show significant differences (Table 2). However, despite the stable bias of males (Table 2) during each season, the trend of the M:F ratio varied, remaining fairly stable during spring–summer (Figure 2B′, C′) and decreasing in colder seasons (Figure 2A′, D′).

The data we obtained for domestic ducks generally fits into the general trend for their wild counterparts. According to a number of studies, sex ratios in waterfowl are often male‐biased (Blums and Mednis 1996; Lehikoinen, Christensen, et al. 2008; Lehikoinen, Öst, et al. 2008). As a result of the research on eider ducks ( *Somateria mollissima* ) over 9 years, Ramula et al. (2018) noted that on average the sex ratio at hatching was slightly female‐biased (52.8%), while in our observations (2014–2020), the sex ratio was male‐biased (51.5%). However, in earlier studies conducted by the same group of authors (Lehikoinen, Christensen, et al. 2008), it was noted that the sex ratio of juvenile ducklings in the wintering areas was male‐biased (~57% males). Although keeping ducks in relatively comfortable farm conditions is far from natural incubation in the northern regions, the results of winter sex ratio shift in our experiments also demonstrated the male bias amounting to 51.2%. A similar prevalence of males over females (~52%) was observed as an average for all seasons.

As a result, we came to a conclusion about the controversial issue of the seasonal effect on the sex ratio in domestic ducks, both when analyzing annual changes and summarizing data for the entire 7‐year period of observations.

**FIGURE 3:**
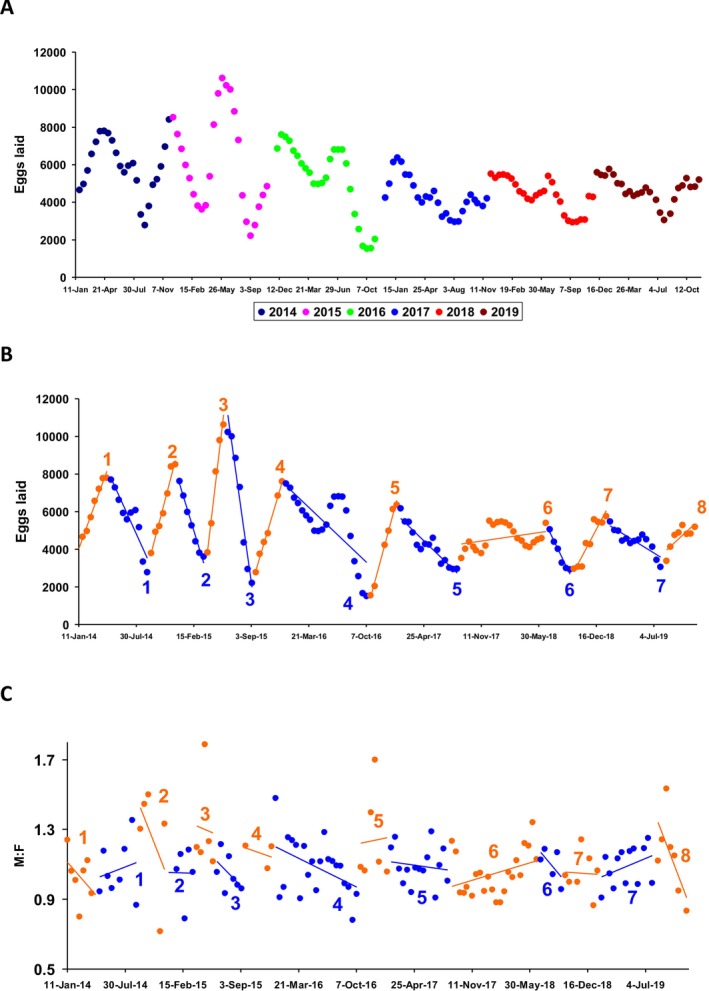
Graphic visualization of changes in (A) egg production during the investigated period (January 11, 2014, to November 23, 2019); (B) egg production splitting the investigated period into the cycles of the oviposition growth (HS) (orange dots and trend lines), and the production drop (LS) (blue dots and trend lines); and (C) M:F ratio according to the cycles of the oviposition growth (HS) (orange dots and trend lines) and drop (LS) (blue dots and trend lines).

The inclusion of data on the number of eggs laid by the duck population in the analysis led us to propose that the breakdown by year and season may not be as successful as previously thought. Due to an incomplete set of egg production data for 2020, we had to exclude this data from the overall analysis (Figure 3A).

### 3.2 Oviposition Cyclicity and Its Relationship With Sex Ratio

Analyzing Figure 3A, we suggested that worthy criteria for a comparison of different parameters within and between the years can be the cycles of growing and dropping in egg production without paying attention to the years themselves.

We analyzed the obtained differences, attempting to identify periods of stable growth and, correspondingly, declines in productivity. Transitions from growth to decline and vice versa were determined by the difference in values between two adjacent points, resulting in a change in sign. Potential outliers within the cycle were accounted for as follows. Ducks are a seasonal species, laying eggs for approximately 6 months (e.g., Lewko and Gornowicz 2025). Thus, a 6‐month productivity period includes a cycle of growth and decline in egg production. Each flock has its own nuances, including both technological features of maintenance and stock renewal, and genetic factors. Nevertheless, it can be suggested that each cycle cannot last less than 3 months. Therefore, we assumed that if a sudden change in the cycle lasted less than 3 months, such a period was considered a random outlier. As a result, we split the data on Figure 3A into the cycles as seen in Figure 3B. The orange dots and their trend lines corresponded to the cycles of the growing oviposition named as “High season” (HS), while the blue dots showed the periods of the egg production drop named as “Low season” (LS).

Thus, we found eight HS cycles and seven LS cycles. The results of the sex ratio shift among ducklings for each cycle are shown in Table 3. Despite the obvious differences in M:F ratios between the HS and LS cycles, no single pair of values, including the averages for the entire observation period, differed significantly.

**TABLE 3:** Data on the ratio of males (M) to females (F) depending on the laying cycle (HS vs. LS).

Cycle	M:F (HS)	M:F (LS)
1	1.02	1.00
2	1.26	1.03
3	1.24	1.06
4	1.13	1.05
5	1.14	1.06
6	1.03	1.12
7	1.04	1.07
8	1.18	
**Average**	**1.08**	**1.05**

In the visualized graphical representation of trends in M:F changes (Figure 3C), it was also quite difficult to trace the HS and LS cyclicity similar to that presented in Figure 3B. During the first two HS cycles, there seemed to be a tendency for the increased number of females in comparison with males and, conversely, an elevation in the number of males in the offspring during LS periods. In the subsequent cycles, this trend changed and reversed, but again became similar to cycles 1–2 by cycles 7–8.

**FIGURE 4:**
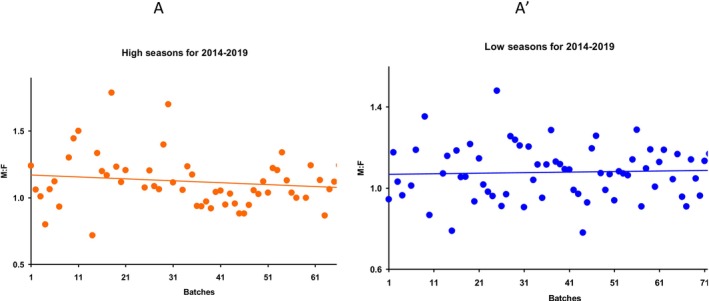
Cumulative data on the M:F ratio during the HS (A) and LS (A′) cycles over the entire observation period (2014–2019).

If we considered the data for all HS and LS periods, combining batches of ducklings obtained into a single numerical series (Figure 4), there was a weak tendency toward a decrease in the M:F ratio (i.e., the tendency of ducks to produce more females) during an HS period and, conversely, slightly more males during an LS cycle. Nevertheless, for both the HS and LS cycles, the quantity of males hatched outnumbered that of females.

**FIGURE 5:**
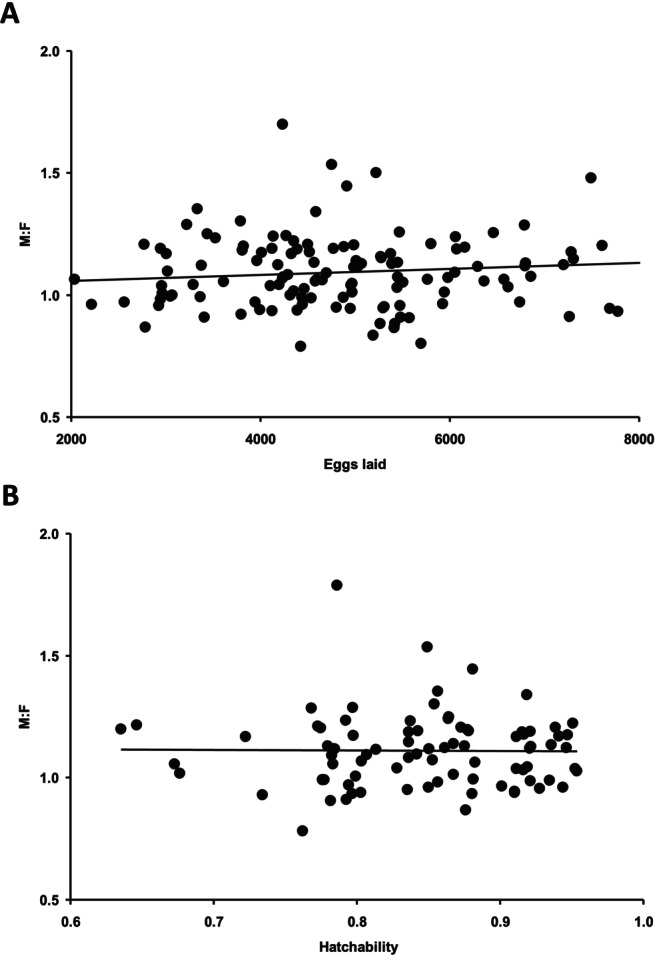
Visualization of the relationship (A) between M:F ratio and duck parent flock performance, and (B) between the M:F ratio and the hatchability level of ducklings.

To resolve the issue of a possible relationship between the value of the M:F ratio and the performance of the parent duck flock, we determined the correlation between these indicators that conformed to *R* = 0.129. Although the resulting *R* value was not significant, a visualization of this relationship (Figure 5A) indicated a weak trend toward an increase in the percentage of males among ducklings hatched.

Another possible reason for the sex ratio bias may be the hatchability level of ducklings (e.g., Batellier et al. 2004; Alonso‐Alvarez 2006). In this respect, we decided to analyze the correlation between the M:F ratio and egg hatchability (as visualized in Figure 5B). Analysis in Figure 5B evidenced the complete absence of this relationship, which was also confirmed by the value of the correlation coefficient *R* = −0.013 that, as is quite expected, is not significant.

Thus, neither seasonal changes throughout the year nor the cyclicity of egg laying were critical to assert their direct impact on the M:F ratio in hatching ducklings. It was likely that there were some other conditions that influence the magnitude and trend of sex ratio alteration. As shown by the analysis of published studies discussed in the Introduction section, a possible influential factor may be weather conditions that in some way affect the parent duck flock. Therefore, we included the examination of such a relationship as one of the goals of the present study. In this regard, the obtained observation results were analyzed based on the premise of weather effect on the parent duck flock.

### 3.3 Influence of Weather Conditions on Sex Ratio and Other Productive Traits

When analyzing the possible effects of weather factors, the dew point closely correlated with *t* (*R* = 0.983, *p* < 0.01) and the gust wind with *w* (*R* = 0.553, *p* < 0.01). In this respect, indicators *t*, *w*, and *P* were chosen for further analysis. In the same way as was undertaken in the previous subsection, we decided to carry out the analysis by (i) separating HS and LS periods and (ii) combining these data. First of all, we assessed how different the average weather data characteristic of the HS and LS cycles are from each other. The results are shown in Table 4.

**TABLE 4:** Mean values of the weather factors for the periods of the growing (HS) and decreasing (LS) oviposition and correlations between them and duck flock performance traits.

Weather factors	HS			LS		
Mean ± SD						
Average temperature, °C	9.5 ± 3.5			10.4 ± 3.9		
Wind speed, km/h	19.4 ± 3.7			19.6 ± 3.8		
Sea level pressure, hPa	1005.1 ± 4.0			1003.9 + 7.2		
**Correlation between weather and performance**						
	**M:F**	**Eggs laid**	**Hatchability**	**M:F**	**Eggs laid**	**Hatchability**
Average temperature, °C	0.215	−0.367^a^	−0.019	−0.099	−0.300^a^	0.017
Wind speed, km/h	−0.355^a^	0.199	−0.006	−0.055	0.295^a^	−0.414^a^
Sea level pressure, hPa	0.273^a^	−0.138	−0.364^a^	−0.029	−0.110	0.223^a^
**Correlation between weather and performance for HS and LS together**						
	**M:F**	**Eggs laid**		**Hatchability**		
Average temperature, °C	0.056	−0.331^a^		−0.014		
Wind speed, km/h	−0.222^a^	0.250^a^		−0.258^a^		
Sea level pressure, hPa	0.168^a^	−0.124		−0.088		

^a^ Correlation coefficients are statistically significant (*p* < 0.05).

None of the mean factor values in Table 4 differed significantly; thus, we may consider that the ducks were under the same weather conditions during both cycles HS and LS. After this, we carried out a correlation analysis of possible relationships between weather conditions and key productivity indicators (as also shown in Table 4).

**FIGURE 6:**
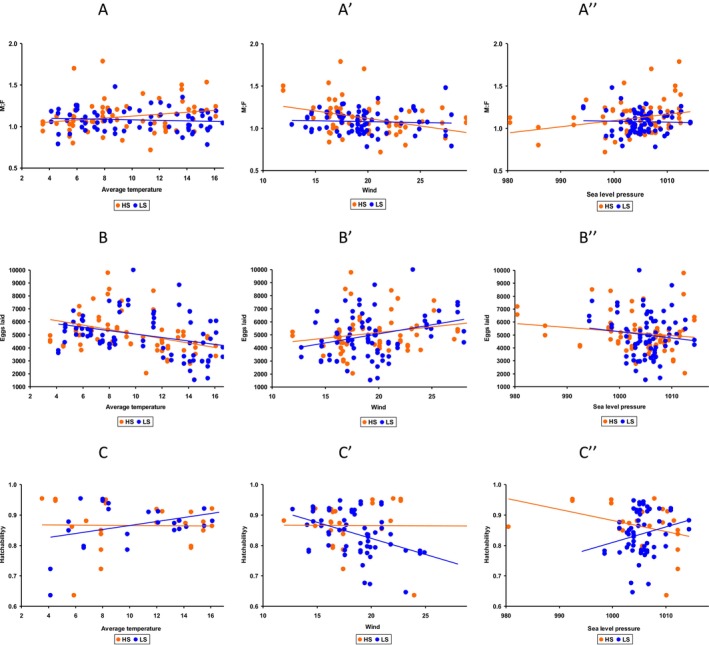
Dependencies of the weather factors, i.e., average temperature (A, B, C), wind (A′, B′, C′), and sea level pressure (A″, B″, C″), on the duck flock traits: the M:F ratio (A, A′, A″), egg production (B, B′, B″), and egg hatchability (C, C′, C″).

Visualization of data in the form of graphical dependencies and trends in their changes (Figure 6) was helpful in coming to more informed conclusions.

Based on the data in Table 4 and Figure 6, we were able to assume that the analyzed meteorological data influenced the main productivity indicators of the parent duck flock. Hereby, the weather affected the sex ratio of hatching ducklings only during HS periods. At the same time, decline LS was characterized by a flat M:F trend, although it was higher than 1 (1.08), approximately corresponding to 51.3% of males. HS cycles could be characterized by the tendency of ducks to increase male production as *t* and *P* increased. However, an increase in *w*, on the contrary, led to the increased egg lay, resulting in a larger number of females.

As for the number of eggs laid, the trends are completely the same, during both HS and LS periods. Both an increase in *t* (Figure 6B) and *P* (Figure 6B″) negatively affected this indicator. However, stronger winds contribute to an increase in the number of eggs laid (Figure 6B′).

With regard to the weather effect on the hatchability level, the situation was quite ambiguous, at least in the case of dividing the results into HS and LS cycles. The wind speed *w* had a negative effect on hatchability during LS periods, remaining unchanged during HS periods (Figure 6C′). Significant differences for both HS and LS cycles were recorded due to the *P* effect (Figure 6C″). However, relatively small changes in this indicator and a large spread in the obtained hatchability values did not enable one to draw unambiguous conclusions. Because of that, it was obviously advisable to consider both cycles (HS and LS) together.

Correlation analysis of the dependence of productivity indicators, i.e., M:F ratio, number of eggs produced and hatchability, based on the combined data for both cycles, are presented in Table 4.

Analyzing the magnitude of the sex ratio shift (M:F), it can be argued that the general trend corresponded to the HS cycle discussed above (Figures 6A, A′, A″). In principle, this pattern was expected, given that the LS cycle was characterized by a constant, unchanged result. This once again demonstrated the advisability of assessing the indicator of sex ratio alteration taking into account the period of HS or LS.

The results in terms of the trend in the parent stock productivity were also expected. Almost identical trend lines when divided into cycles (Figures 6B, B′, B″), naturally, showed similar trends when combining the results of observations of this indicator. However, the situation changed somewhat for the hatchability indicator. Its relationship with *P* was very small (−0.088) and, accordingly, insignificant, which was quite logical, given the opposite directions of the trend lines when dividing the data into HS and LS cycles (Figure 6C″). At the same time, the trend with a negative effect of *w* on hatchability remained when combining the results of both cycles. In addition, it is obviously more appropriate to assess the influence of this weather factor by considering the division into HS and LS (Figure 6C′).

The results obtained by assessing the relationship between the main indicators of the parent duck flock led us closer to a possible solution for the problem of changing the sex ratio artificially. Based on the data shown in Figures 6A, A′, A″, it was possible to operate with this parameter by changing *t*, *w*, and *P*, but only during HS periods. If we reject the factor of changes in *P* as unrealistic, lower *t* and increased *w* can lead to an increased percentage of females, while an increase in *t* during relative calm *w*, on the contrary, resulted in a male bias (Figures 6A, A′). Hereby, it should be taken into account that an artificial change in these meteorological parameters can also affect the number of eggs laid, which, if we want to shift the M:F ratio in favor of females, can lead to negative consequences in egg production (Figures 6B, B′).

The question regarding the *w* effect remains open. If its strengthening seems to be a completely logical factor for reducing hatchability, its effect on egg production growth is not entirely amenable to logical explanation. Although *w* did not affect reproductive behavior in mandarin ducks in the study of Munday and Rose (2022), its influence was most likely offset in our studies by the fact that during strong winds the ducks hid in the poultry house.

We also decided to analyze the possible effect of multiple correlation from the impact of weather factors on the parameters of our interest, i.e., M:F, number of eggs laid (*N*), and hatchability (*H*). Based on a comparative analysis of the feasibility of various multivariate models (Koper and Manseau 2009; Grueber et al. 2011; Pekár and Brabec 2018), a choice was made in favor of linear multiple regression equations. This choice was based on the fact that data series of dependent variables were normally distributed. That was confirmed by the Shapiro–Wilk test (Shapiro and Wilk 1965). Calculation according to Equation 2 showed *W* values of 0.998 for M:F; 0.999 for *N*; and 0.999 for *H* with a tabulated value of *W*_*t*_ = 0.970 (Royston 1982; Royston 1992).

As a result, the following regression equations were obtained for the corresponding productivity cycles:

‐ Periods of the growing oviposition (HS)

(3) M:F=−0.736−0.0006t−0.0156w+0.0022P

with *R* ^2^ = 0.131 (*R* = 0.361^a^)

(4) N=30147.94−199.23t−55.33w−21.94P

with *R* ^2^ = 0.142 (*R* = 0.377^a^)

(5) H=11.741−0.012t−0.024w−0.01P

with *R* ^2^ = 0.394 (*R* = 0.628^a^).

‐ Periods of the decreasing oviposition (LS)

(6) M:F=5.8013−0.0071t−0.009w−0.0045P

with *R* ^2^ = 0.038 (*R* = 0.196)

(7) N=−11636.21−84.41t+95.18w+15.59P

with *R* ^2^ = 0.113 (*R* = 0.336^a^)

(8) H=1.2514−0.0056t−0.0143w

with *R* ^2^ = 0.228 (*R* = 0.477^a^).

Multiple correlation indicates some progress in the calculated prediction of all assessed indicators (M:F, *N*, and *H*) from the complex impact of weather data. The increase in *R* values was also accompanied by an increase in their statistical significance. The significance of *R* was noted for all equations, except for the calculation of the M:F ratio (Equation 6) in the LS period. However, the values of the determination coefficients (*R* ^2^), by the value of which it is possible to judge the quantitative ratio of the correct prediction, are not very optimistic. The most accurate result of calculating the *H* value in HS (Equation 5) demonstrated that with the help of weather factors *t*, *w*, and *P*, it is possible to correctly predict only slightly less than 40% of the data. The prediction of the remaining indicators was even lower: 13% for M:F (Equation 3) and 14% for *N* (Equation 4) in the HS period. For the LS period, the prediction results were even lower.

Thus, it can be concluded that the weather factors expressed by the values of *t*, *w*, and *P* have a small but significant impact on the studied M:F, *N*, and *H* indices, especially during the period of productivity growth. However, there are other indices that influence both the sex shift and a number of other productive parameters that are obviously not related to weather conditions and their identification was not successful within the framework of these studies.

## 4 Conclusions

We herein report the results of observations of the parent duck flock and their performance indicators, i.e., egg production, hatchability, and sex ratio of ducklings after hatching. These findings reflect the secondary sex ratio, which may reflect sex‐biased embryonic mortality rather than a shift in the primary sex ratio. Therefore, the results obtained will be more useful for poultry industry than for studying the biological processes of avian reproduction. Analytical processing of the results obtained made it possible to assume the following postulates relevant to reproductive physiology and behavior in domestic ducks:

1. When analyzing a 7‐year observation of the parent duck flock, we came to the conclusion that it was advisable to divide the observation intervals not by seasons, but by periods of productivity, i.e., cycles of growth and decline in oviposition. This approach allowed us to obtain more adequate results and come to substantiated conclusions about the various levels of influence of environmental factors on the studied indicators.
2. Weather factors to which the parent flock of ducks was exposed have a low but significant impact on productivity indicators. At the same time, the sex ratio shift in hatching ducklings turned out to be significant under the influence of *t*, *w*, and *P* only during HS periods of their mothers. During LS periods, ducks proved to be unaffected by meteorological factors, consistently producing just over 51% males.
3. The egg production of the parent duck flock directly depended on weather variability during any oviposition period. At the same time, a decline in egg production was observed with increasing *t* and *P*. On the other hand, stronger winds had a beneficial effect on the number of eggs laid.
4. The factor of *w* to which the duck population was exposed also influenced the subsequent hatchability of their eggs, but only during LS periods, significantly reducing this indicator with increased gusts. Taking into account the data from the previous point (3), we suggest that this phenomenon was associated with an increase in egg production under the influence of the same weather parameter. Perhaps an increase in the number of eggs laid led to a decrease in their quality indicators, which, in turn, negatively affected the hatchability rate.
5. It is possible that, if we receive confirmation of the dependences and relationships we have obtained, in the future, by creating artificial weather conditions for ducks, it would be possible to achieve a shift in the sex ratio of ducklings by regulating *t* and *w*, depending on the need for production of females or males. However, one should also take into account the factor of a possible change in the number of eggs laid due to the influence of the same weather characteristics. Nevertheless, given the modest magnitude of observed effects, the low predictive power, and possible trade‐offs with egg production and hatchability, this claim should be proved with further results.

## Author Contributions

**Valeriy G. Narushin:** conceptualization, data curation, formal analysis, investigation, methodology, resources, software, visualization, writing – original draft, writing – review and editing. **Michael N. Romanov:** project administration, validation, writing – review and editing. **Sabine Klein:** investigation, validation, writing – review and editing. **Attila Salamon:** investigation, validation, writing – original draft, writing – review and editing, project administration. **Gianluca Manzoni:** investigation. **Darren K. Griffin:** validation, writing – review and editing. **John P. Kent:** investigation, validation, writing – review and editing.

## Funding

The authors have nothing to report.

## Conflicts of Interest

The authors declare no conflicts of interest.

## Data Availability

Data are available from the Figshare data repository, https://figshare.com/s/90e982ea27520c637874.
